# Oxidative Stress in Melanoma: Beneficial Antioxidant and Pro-Oxidant Therapeutic Strategies

**DOI:** 10.3390/cancers15113038

**Published:** 2023-06-02

**Authors:** Alyssa L. Becker, Arup K. Indra

**Affiliations:** 1 Department of Pharmaceutical Sciences, College of Pharmacy, Oregon State University (OSU), Corvallis, OR 97331, USA; beckera3@hawaii.edu; 2 John A. Burns School of Medicine, University of Hawai’i at Mānoa, Honolulu, HI 96813, USA; 3 Knight Cancer Institute, Oregon Health & Science University (OHSU), Portland, OR 97239, USA; 4 Department of Biochemistry and Biophysics, Oregon State University (OSU), Corvallis, OR 97331, USA; 5 Linus Pauling Science Center, Oregon State University (OSU), Corvallis, OR 97331, USA; 6 Department of Dermatology, Oregon Health & Science University (OHSU), Portland, OR 97239, USA

**Keywords:** melanoma, oxidative stress, ROS, antioxidants, NRF2, thioredoxin reductase, glutathione, superoxide dismutase, BRAF/MEK inhibitors, N-acetylcysteine (NAC)

## Abstract

**Simple Summary:**

Cutaneous melanoma is one of the deadliest forms of skin cancer. While advancements in systemic targeted therapies and immunotherapies have greatly improved melanoma survival in recent years, tumor resistance can limit the efficacy of these therapies. Targeting redox homeostasis in melanoma progression is a promising therapeutic approach, especially in cases of melanoma drug resistance. The role of oxidative stress in melanoma is paradoxical in that it promotes tumor initiation but prevents vertical growth and metastasis. As the disease progresses, melanoma employs adaptive mechanisms to decrease oxidative stress in the tumor environment. Thus, agents with antioxidant properties may have the greatest utility in chemoprevention whereas those with pro-oxidant properties may be better suited for treatment. The purpose of this review is to provide an overview of oxidative stress in melanoma, and how the antioxidant system may be manipulated in a therapeutic context for improved efficacy and survival.

**Abstract:**

Cutaneous melanoma ranks as the fifth most common cancer in the United States and represents one of the deadliest forms of skin cancer. While recent advances in systemic targeted therapies and immunotherapies have positively impacted melanoma survival, the survival rate of stage IV melanoma remains at a meager 32%. Unfortunately, tumor resistance can impede the effectiveness of these treatments. Oxidative stress is a pivotal player in all stages of melanoma progression, with a somewhat paradoxical function that promotes tumor initiation but hinders vertical growth and metastasis in later disease. As melanoma progresses, it employs adaptive mechanisms to lessen oxidative stress in the tumor environment. Redox metabolic rewiring has been implicated in acquired resistance to BRAF/MEK inhibitors. A promising approach to enhance the response to therapy involves boosting intracellular ROS production using active biomolecules or targeting enzymes that regulate oxidative stress. The complex interplay between oxidative stress, redox homeostasis, and melanomagenesis can also be leveraged in a preventive context. The purpose of this review is to provide an overview of oxidative stress in melanoma, and how the antioxidant system may be manipulated in a therapeutic context for improved efficacy and survival.

## 1. Introduction

Over the past 30 years, the global incidence of cutaneous melanoma has consistently risen, and projections by the International Agency for Research on Cancer suggest that this trend will persist [1]. In the United States, melanoma is the fifth most common cancer among both men and women [2]. According to the American Cancer Society, in 2023, an estimated 97,610 adults (58,120 men and 39,490 women) in the United States will be diagnosed with invasive cutaneous melanoma. Worldwide, an estimated 324,635 people were diagnosed with melanoma in 2020 [2]. While cutaneous melanoma accounts for the majority of skin-cancer related deaths, the 5-year survival rate is quite favorable (98–99%) when treated in its earliest stages [3,4]. In contrast, the 5-year survival rate of stage IV melanoma is approximately 32% [3]. Due to the association between advanced disease and unfavorable patient outcomes, early detection and effective therapeutic approaches have been vital to improving melanoma survival in recent years [5].

Melanoma develops from the malignant transformation of melanocytes, which are dendritic pigment-producing cells located in the basal layer of the epidermis, hair follicles, inner ear, and uvea of the eye [6,7,8]. Cutaneous melanoma is characterized by a high mutational burden and is frequently associated with mutations in genes that regulate cell proliferation and survival, including BRAF, NRAS, NF1, and tumor suppressor PTEN [9,10,11,12,13,14,15]. These mutations lead to overactivation of the mitogen-activated protein kinase (MAPK) pathway and phosphatidylinositol 3-kinase (PI3K)/protein kinase B (AKT) signaling pathways. Currently, the most common genetic cause of melanoma is the BRAFV600E mutation, which is present in approximately 50% of melanomas, and contributes to increased cell proliferation and metabolic reprogramming [10,16,17]. Other genetic alterations have been identified in cutaneous melanoma, including mutations in cell cycle regulators like CDK4, and tumor suppressor genes TP53 and CDKN2A [11,12,13,18]. These mutations contribute to alterations in telomere maintenance, histone modification and methylation, which affect gene expression and contribute to the development and progression of the disease. 

Current therapeutic interventions for cutaneous melanoma include surgical resection, radiation, chemotherapy, immunotherapy, and targeted therapy [19]. Surgical resection of the tumor with wide margins is curative in early-stage melanoma localized to the skin. Of note, surgical excision may be also be used to treat accessible solitary metastatic lesions; in the case of several metastatic tumors, radiation therapy may be employed, especially in lesions affecting the skin, bone, or brain [20]. Systemic therapies are typically reserved for advanced melanoma (stage III and IV) following surgical resection. Earlier untargeted chemotherapies (e.g., dacarbazine and temozolomide) have largely been replaced by more effective immunotherapy and targeted therapy agents, which exploit the tumor microenvironment [21]. Immunotherapy drugs have revolutionized melanoma treatment and significantly improved outcomes for patients with advanced or metastatic disease [22,23,24,25]. This category includes immune checkpoint inhibitors like pembrolizumab (anti-PD-1), nivolumab (anti-PD-1), ipilimumab (anti-CTLA-4), and cytokine therapies like interleukin-2 [26]. Approved targeted therapies for melanoma, which may be used if the cancer progresses despite initial immunotherapy treatment and in patients with BRAF-mutations, include BRAFV600E and MEK inhibitors (e.g., vemurafenib, dabrafenib, trametinib) [27]. Despite the variety of systemic treatment options available for metastatic melanoma, efficacy may be limited by tumor resistance [28,29].

Targeting redox homeostasis in melanoma progression is a promising therapeutic approach, especially in cases of melanoma drug resistance [10]. The purpose of this review is to provide an overview of oxidative stress in melanoma, and how the antioxidant system may be manipulated in a therapeutic context for improved efficacy and survival.

## 2. Oxidative Stress in Melanomagenesis

Oxidative stress refers to an imbalance between the production of reactive oxygen species (ROS), reactive nitrogen species (RNS) and the antioxidant defense mechanisms that neutralize them [30]. ROS are highly reactive molecules that can cause damage to cellular macromolecules, such as lipids, proteins, and DNA, if they accumulate to excessive levels. Endogenous sources of ROS include nicotinamide adenine dinucleotide phosphate hydrogen (NADPH) oxidase (NOX), cyclooxygenases, lipooxygenases, and cytochrome P450, endothelial nitric oxide synthase (eNOs) and others. Exogenous sources of ROS include UV irradiation and inflammation [31,32]. Biomarkers for oxidative stress include DNA lesions in the form of 8-hydroxy-2′-deoxyguanosine (8-OHdG), end products of lipid peroxidation, malondialdehyde (MDA) and 4-hydroxy-2-nonenal (HNE), and derivatives of protein oxidation, advanced oxidation protein products (AOPPs) and advanced glycation end products (AGEs). 

Under normal conditions, the antioxidant response system maintains a balance between ROS production and scavenging. The antioxidant response includes non-enzymatic and enzymatic molecules: glutathione (GSH), which belongs to the glutathione system, also involving glutathione reductase, glutathione peroxidase (GPX) and glutathione-s-transferase (GST), superoxide dismutase (SOD), superoxide reductase, catalase (CAT) and thioredoxin (TRX) [33,34]. However, under certain conditions, such as exposure to UV radiation, environmental toxins, or inflammation, the production of ROS can overwhelm the antioxidant defenses and lead to oxidative stress.

Oxidative stress and redox homeostasis are implicated in all phases of melanomagenesis as well as in the emergence of drug resistance [10,35]. The role of oxidative stress is somewhat paradoxical: while ROS help to promote cancer survival, proliferation, tumor vascularization and metastasis, at high levels, they can also cause DNA damage and cancer cell death [10,35,36,37]. Thus, melanoma requires adaptive strategies to resist the effects of increased ROS levels [38,39]. Various studies have investigated the antioxidant response of melanoma cells, and show increased levels of enzymes that protect the tumor from ROS damage [10,35,40,41,42]. GR is associated with oxidative stress regulation in melanoma. Inhibition of GR suppresses lung metastasis and subcutaneous growth of melanoma, while also impacting other cellular behaviors like proliferation, colony formation, migration, and invasion [43]. GPX1 expression increases with melanoma progression, correlating with heightened proliferation [44]. GPX3, on the other hand, can act as a tumor suppressor gene and is typically downregulated in various tumors, including melanoma [45,46]. Low GPX3 levels are linked with higher proliferation, motility, invasiveness, and poor prognosis [45]. GST levels decrease in metastasis derived from skin or lymph nodes but increase with overall tumor progression. GST is directly involved in melanoma invasion and the development of drug resistance, with GST π being the most expressed isoform [47,48]. However, GSTT and GSTM isozymes are absent in a large population portion and could influence melanoma development by decreasing oxidative stress [49]. The role of SOD2 in melanoma is controversial, with some studies linking lower levels to metastasis [50]. High SOD2 expression can suppress melanoma’s malignant phenotype in vitro, but serum SOD2 levels may increase in melanoma patients and correlate with disease progression [51]. Alterations in SOD expression also relate to therapy resistance. SOD1 is involved in melanogenesis and/or differentiation, while SOD3 overexpression inhibits the growth of specific melanoma cells [36,52]. In melanoma, there appears to be a redox imbalance characterized by decreased catalase activity and increased SOD activity [53]. Catalase activity may increase in stages I, II, and III, but not in stage IV, suggesting a protective role against metastasis [54]. Mutations in the CAT gene were found in 10% of the melanoma patients, mostly leading to increased mRNA expression, but these mutations had no significant impact on survival [34]. Transcriptional factors like Ap-1 and NF-kB and activation of the MAPK pathway and S-phase kinase-associated protein 2/Skp2/MTH1 axis have been implicated in protecting melanomas from ROS damage [55,56]. Overexpression of ecto-enzyme gamma-glutamyltransferase (GGT) and CD 147, a cell surface receptor for cyclophilin A, have also been shown to be involved in melanoma’s resistance to oxidative stress [57,58,59]. The aldehyde dehydrogenases convert toxic aldehydes into non-toxic carboxylic acids. Upregulation of ALDH1 isozymes have been reported in several malignancies. ALDH1A1 has been implicated in tumorigenicity, invasion, and resistance to chemotherapy, in various types of cancer, including melanoma [60]. Whereas ALDH1B1 has also been demonstrated to be upregulated in several cancers, acting as an oncogene, but its role in melanoma has not been determined [61,62]. Conversely, ALDH2 expression has been shown to be downregulated in melanoma, leading to acetylaldehyde accumulation, which results in poorer prognosis [63,64]. Zhai et al. analyzed gene profiling datasets GSE15605, GSE7553, and GSE46517, to compare the gene expression levels of ALHD1A1, ALDH1B1, and ALDH2 in normal human skin versus metastatic melanoma [65]. They found that ALDH2 expression was significantly downregulated in 3 Gene Expression Omnibus (GEO) melanoma datasets. Interestingly, while oxidative stress increases in metastasizing cells, it limits their ability to invade and metastasize [66]. The low GSH-to- glutathione disulfide (GSSG) ratio seen in metastatic melanoma suggests that metastatic cells attempt to maintain redox homeostasis by consuming GSH. Isocitrate dehydrogenase (IDH2) is one of the key enzymes in melanoma’s antioxidant system, and downregulation of IDH2 has been shown to inhibit tumor growth [67]. Additionally, Nuclear factor erythroid-2-related factor (NRF2), which is one of the most essential proteins involved in regulating the antioxidant response, modulates the expression of antioxidant enzymes HO-1 (heme oxygenase-1), SOD, CAT, and GPX, and can have both an onco-promoter and onco-suppressor function depending on the context [10,68,69]. Normal expression of NRF2 has a cytoprotective role against initial melanomagenesis, and NRF2 depletion and ROS accumulation is associated with cancer initiation. However, aberrant overexpression of NRF2 increases antioxidant responses and changes the cellular redox state, aiding in melanoma cell survival, growth, and metastasis [70]. According to a recent study by Davalos et al., Nuclear Receptor Subfamily 2 Group F, Member 2—isoform 2 (NRF2F2-Iso2) promotes the progression of metastatic melanoma by influencing the activity of full-length NR2F2 on neural crest cell (NCC) and epithelial-to-mesenchymal transition (EMT) target genes [71]. Importantly, NRF2 may promote invasion and migration by upregulating HO-1 [72]. NRF2 has also been shown to upregulate P62, another driving force in tumor growth and metastasis [73,74]. NRF2 and P62 are linked through a positive feedback loop: NRF2 directly promotes P62, and interactions between P62 and Keap1 (Kelch-like ECH-associated protein 1) prevent Keap1 from inhibiting NRF2. Because constant NRF2 activation promotes tumor development and metastasis, it is thus considered a negative prognostic factor and associated with worsened melanoma-specific survival [68,75,76]. A positive correlation has been demonstrated between NRF2 expression, Clark level, Breslow index and nodular development [69]. Whereas expression of Calpain-3, an intracellular cysteine protease that increases formation of ROS, is downregulated in the vertical growth phase and metastasis [77]. Metallothioneins (MTs) are a family of small, cysteine-rich proteins that are involved in metal homeostasis and detoxification [78,79]. In melanoma, MTs are upregulated in response to oxidative stress. They are capable of binding and sequestering metal ions, such as zinc and copper, which are essential for the catalytic activity of ROS-producing enzymes like NADPH oxidase [79,80]. Furthermore, MTs possess antioxidant properties themselves, as they can scavenge ROS directly, protecting melanoma cells from oxidative damage induced by ROS [79]. Heat Shock Protein 27 (HSP27) functions as a molecular chaperone and assists in protein folding, preventing the aggregation and denaturation of proteins under stressful conditions [81,82]. Additionally, HSP27 can interact with other proteins involved in apoptosis pathways, such as caspases, and inhibit their activation [82,83]. Of note, the antioxidant system response has also been implicated in disease resistance to targeted therapy [59,84]. 

It should be recognized that the mechanism and impact of melanin on oxidative stress in the context of melanomagenesis has not been fully elucidated. While the production of melanin can result in oxidative stress, once formed, the melanin pigment has unpaired electrons and can interact with free radicals and other reactive species as an antioxidant [85,86]. However, as Slominski et al. points out, this is a double-edged sword: shielding healthy melanocytes from oxidative stress but potentially accelerating disease and limiting the response to therapies that induce oxidative stress [7]. One study demonstrated that induction of melanogenesis in amelanotic human and hamster melanoma cells resulted in significant transformations in the cells’ metabolic condition and performance, both biochemically and molecularly [87]. These shifts were paired with a notable surge in hypoxia inducible factor (HIF-1α) expression in the nuclear components derived from pigmented cells. Thus, it can be inferred that pigmented melanomas may be more resistant to therapies that induce oxidative stress compared to amelanotic melanomas. However, it’s important to note that the response to oxidative stress is influenced by a number of factors. 

## 3. Preventive and Therapeutic Strategies That Target Oxidative Stress Pathways

**Figure 1:**
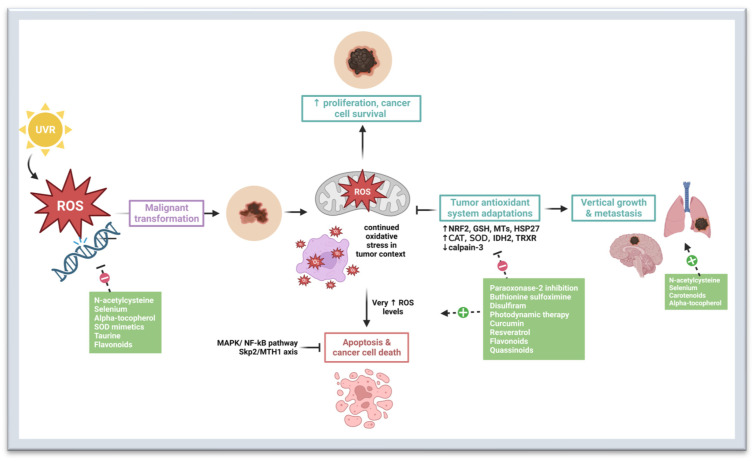
Oxidative Stress and Therapeutic Targets in Melanoma. Oxidative stress refers to an imbalance between the production of reactive oxygen species (ROS) and reactive nitrogen species (RNS) and the antioxidant defense mechanisms that neutralize them [30]. Exogenous sources of ROS include UV irradiation and inflammation [31,32]. Endogenous sources of ROS include nicotinamide adenine dinucleotide phosphate hydrogen (NADPH) oxidase (NOX), cyclooxygenases, lipooxygenases, cytochrome P450, endothelial nitric oxide synthase (eNOs), and others. Oxidative stress and redox homeostasis are implicated in all phases of melanomagenesis [10,35]. The role of oxidative stress is somewhat paradoxical: while ROS help to promote cancer survival, proliferation, tumor vascularization, and metastasis, at high levels, they can also cause DNA damage and cancer cell death [10,35,36,37]. Thus, melanoma requires adaptive strategies to resist the effects of increased ROS levels [38,39]. N-acetylcysteine, selenium, alpha-tocopherol, SOD mimetics, and flavonoids have antioxidant properties that may be chemo-preventive in utility [89,90,91,92,93,94,95]. However, studies have shown that antioxidants such as n-acetylcysteine, selenium, carotenoids, and alpha-tocopherol can promote growth and metastasis in later diseases by bolstering the tumor’s antioxidant system adaptations [91,96,97]. Paraoxonase-2 inhibition, buthionine sulfoximine, disulfiram, photodynamic therapy, curcumin, resveratrol, flavonoids, and quassinoids inhibit tumor antioxidant system adaptations through various mechanisms, leading to increased oxidative stress and cancer cell death [35,98,99,100,101,102,103,104,105,106,107,108,109,110,111,112,113,114,115,116]. Abbreviations: UVR—ultraviolet radiation; ROS—reactive oxygen species; MAPK—mitogen-activated protein kinase; NF-kB—nuclear factor kappa B; Skp2—S-phase kinase associated protein 2; MTH1—MutT homolog 1; NRF2—nuclear factor erythroid 2-related factor 2; GSH—glutathione; CAT—catalase; SOD—superoxide dismutase; IDH2—isocitrate dehydrogenase 2; TRXR—thioredoxin reductase; MTs—metallothioneins; HSP27—Heat Shock Protein 27. Created with BioRender.com.

Burgeoning preventive and therapeutic approaches to melanoma exploit the complex interplay between oxidative stress, redox homeostasis, and melanomagenesis. Given the role of ROS in tumor initiation and progression, antioxidants are an interesting potential option for melanoma prevention and treatment. However, later in pathogenesis, melanoma tumors develop antioxidant mechanisms, but ROS levels are still higher compared to normal cells [34,88]. Therefore, further induction of oxidative stress may result in preferential demise of malignant cells. In other words, both antioxidants and pro-oxidants may have utility in the context of melanoma interventions (Figure 1). The till date usage of various small molecules, bioactive compounds and phytochemicals (e.g., polyphenols) with anti-oxidant properties in melanoma prevention and inhibition of disease progression has been described in the following sub-sections. 

### 3.1. N-Acetylcysteine

N-acetylcysteine (NAC) is an affordable, highly potent, orally bioavailable, and cell-permeable antioxidant [89]. One murine study reported that pre-treatment with NAC reduced thiol depletion and blocked formation of 8-oxoguanine in mouse skin following neonatal UV treatment, and significantly delayed mean onset of UV-induced melanocytic tumors [90]. A 2009 study in 72 individuals with a history of numerous or atypical nevi and/or personal or family history of melanoma, concluded that NAC can be safely administered to patients to modulate UV-induced oxidative stress in nevi, suggesting that NAC may be a potential chemo-preventive agent in melanoma [89]. Ultraviolet radiation-induced glutathione depletion was attenuated in the post-drug (compared with pre-drug) nevus in approximately half of patients tested. While the above results seem promising, later studies reported that incubating human melanoma cells with NAC increased cell migration and cell division, and NAC-supplemented drinking water accelerated metastasis in murine models of malignant melanoma [66,117]. A 2022 study in a murine melanoma model reported a potential mechanism for this, demonstrating that high doses of NAC can cause metastatic spread of malignant melanoma, increase the generation of ROS, and increase the nuclear translocation of NRF2 [96]. Altogether, the above results suggest that NAC is the quintessential example of the paradoxical nature of the oxidative stress pathways involved in melanomagenesis and should be used with caution. 

### 3.2. Selenium

Selenium is an essential micronutrient that was first studied in the context of cancer prevention nearly three decades ago [118,119]. The role of selenium supplementation in melanoma prevention and treatment is controversial and its efficacy is dependent on several factors. Cassidy et al. used a combination of in vitro and in vivo models to examine the utility of two unique chemical forms of selenium in these contexts. The results were mixed with topical treatment with l-selenomethionine delaying the time required for UV-induced melanoma development, but increasing the rate of growth of those tumors once they appear [91]. Whereas oral administration of high dose methylseleninic acid significantly decreased the size of human melanoma xenografts. They also found that a modest elevation of selenium levels in the skin may accelerate the growth of developing tumors. Additionally, a systematic review and meta-analysis of nine studies, including, randomized controlled trials and prospective observations studies, showed that the consumption of selenium, vitamin C, E (tocopherol), and A (retinol), carotenoids and selenium, as food, supplements, or both, or high fruit and vegetable intake did not affect the incidence of cutaneous melanoma [120].

### 3.3. Carotenoids

Carotenoids are natural pigments found in various fruits and vegetables that have antioxidant properties [121]. They include β-carotene, lycopene, lutein, and zeaxanthin, among others. Several clinical studies have investigated the relationship between carotenoid intake and melanoma risk. These studies have primarily focused on dietary intake rather than using carotenoids as direct treatments for melanoma. The Vitamins and Lifestyle (VITAL) study, a large prospective cohort of 69,635 participants found that dietary or total intake of carotenoids was not associated with melanoma risk [122]. The VITAL study was unable to detect an association of melanoma risk with regard to lutein and lycopene supplements as the cohort was underpowered for these carotenoids. The Women’s Healthy Study, a randomized, double-blind placebo-controlled trial, also reported that β-carotene intake had no impact on melanoma risk, with a relative risk of 0.90 (95% CI 0.49–1.68) [123]. A meta-analysis of randomized controlled trials found that β-carotene supplementation did not affect the incidence of melanoma (RR, 098; 95% CI, 0.65–1.46) [124]. As noted above, in Miura and Green’s meta-analysis, consumption of carotenoids as food, supplements, or both did not affect the incidence of cutaneous melanoma [120] However, a recent murine study found that β-carotene accelerated metastasis in mice with BRAFv600E-driven melanoma [97]. They also reported that the transcription factor BACH1 is activated following antioxidant administration, and silencing of Bach1 in murine melanoma models resulted in reduced metastasis.

### 3.4. Alpha-Tocopherol

With its capacity to neutralize free radicals and hinder lipid peroxidation, Vitamin E (alpha-tocopherol) demonstrates potent antioxidant properties [125]. However, Miura and Green’s meta-analysis reported that the consumption of vitamin E as food, supplements, or both did not affect the incidence of cutaneous melanoma [120]. Whereas the SU.VI.MAX trial, a randomized, placebo-controlled investigation into the impact of antioxidant vitamins and minerals on health, reported that women consuming antioxidant supplements, including dietary supplementation with vitamin E, were at an increased risk for developing melanoma [126] One in-vivo study showed that pretreatment with topical vitamin E and NAC 24 h prior to UV exposure significantly decreased the expression of matrix metalloprotease-12 in UV-irradiated skin, suggesting a potential protective effect [93]. However, Kashif et al. found that the vitamin E analogue, Trolox, reduced ROS levels and increased migration of melanoma cells [97]. Thus, alpha-tocopherol may have value in a preventive context when applied topically but likely has a negative effect in the active disease state.

### 3.5. Superoxide Dismutase Mimetics

Superoxide dismutase mimetics are a class of compounds designed to replicate the activity of the natural SOD enzymes, and studies have demonstrated their potential in melanoma prevention and adjunct therapy. One study demonstrated that an exogenous manganese superoxide dismutase isolated from *Allium sativum*, a medicinal plant, was capable of modulating intracellular reactive oxygen species levels and inhibiting cell multiplication in tumor cells [94]. A noted decrease in SOD levels during early stages of cancer development implies its potential for preventive measures. While lacking widespread clinical trials and pharmaceutical industry support, SOD liposomes and mimetics have been effective in animal models and early-phase clinical trials [127]. Another therapeutic strategy utilizes GC4419, a selective SOD mimetic in combination with pharmacological ascorbate to enhance the effects of radiation [128]. Lastly, small molecule SOD mimetics have been shown to minimize radiation and chemotherapy-induced normal tissue injury, indicating a further area where SOD might enhance patient outcomes in melanoma therapy [129]. Overall, the role of SOD and its mimetics in influencing intracellular reactive oxygen species, preventing cell multiplication, and mitigating the harmful effects of cancer therapies shows promising avenues for melanoma prevention and treatment.

### 3.6. Taurine

The antitumor and cytoprotective effects of taurine are linked to its ability to enhance antioxidant capacity, boost immunity, synergize with chemotherapeutic agents, and reduce chemotoxicity [130,131]. In B16F10 murine melanoma cells, taurine has been shown to increase the activity of SOD and GPX, leading to a decrease in ROS levels and inhibition of tumor cell growth [95]. Monocarboxylate transporter 7 (MCT7)/Slc16a6, a novel facilitative taurine transporter, has been identified as a survival factor in melanoma [132]. MCT7 is highly expressed in melanoma cells, and two independent single-nucleotide polymorphisms (SNPs), PDSS1 rs12254548 G>C and SLC16A6 rs71387392 G>A, with allelic hazard ratios of 0.58 (95% confidence interval [CI] = 0.44–0.76, *p* = 9.00 × 10^−5^) and 1.98 (95% CI = 1.34–2.94, *p* = 6.30 × 10^−4^), respectively, have been linked to cutaneous melanoma survival [133]. Thus, taurine supplementation or targeting its transporters may have therapeutic potential in melanoma treatment.

### 3.7. Paraoxonase-2 Inhibitors

Another promising therapeutical target in melanoma is the intracellular enzyme, paraoxonase-2 (PON2) [35]. PON2 exerts antioxidative properties within the mitochondrial respiratory chain by binding with high affinity to coenzyme Q10 within the inner membrane, leading to a reduction of superoxide anion release during the electron transport chain [98,134,135]. Interestingly, expression of PON2 has been shown to be positively correlated with Breslow thickness, Clark level, regression, mitoses, lymph node metastases, and thus, overall staging [136]. Because of this, Bachetti et al. assert that PON2 may have utility as a biomarker of tumor aggressiveness in melanoma. Additionally, silencing of the gene responsible for the enzyme in human A375 melanoma cells bearing BRAFV600E mutation has been shown to improve chemosensitivity to cisplatin; thus, paraoxonase-2 inhibitors may be of therapeutic value [98]. 

### 3.8. Buthionine Sulfoximine

Buthionine sulfoximine (BSO) also acts to increase chemosensitivity. There is a growing body of evidence to suggest that depleting antioxidant defenses, particularly GSH levels, which are implicated in chemoresistance to alkylating agents, may prove to be a viable strategy in treatment resistant melanoma [99]. Recent reports have shown encouraging results from combining chemotherapy with buthionine sulfoximine (BSO), a sulfoximine derivative that reduces GSH levels by inhibiting gamma-glutamylcysteine synthetase, the enzyme responsible for the initial step of glutathione synthesis. BSO was found to enhance melphalan cytotoxicity by 2.46-fold in SK-MEL 28 melanoma cells [99]. 

### 3.9. Disulfiram

Disulfiram, an FDA-approved drug for the treatment of alcoholism, is a potent inhibitor of copper-zinc superoxide dismutase. Several mechanisms of its antitumor activity have been suggested, including induction of reactive oxygen species and death signaling pathways [137]. When complexed with copper ions, the metabolite of disulfiram, diethyl dithiocarbamate (DDTC) has also been shown to inhibit the ubiquitin-proteasome system [137,138,139]. It is hypothesized that the antitumor activity may be closely related to lipid peroxidation accumulation and ferroptosis mediated by the SLC7A11/GPX4 signaling pathway [139]. Disulfiram has been shown to improve the chemosensitivity of melanoma cells to oxaliplatin treatment, according to several studies [100,101,102]. Another study on the cytotoxic activity of the combination of curcumin with disulfiram in murine B16-F10 melanoma cells showed that a combination of curcumin and disulfiram displayed synergistic tumor growth inhibition [103]. Although these results are promising, the Cu-DDTC complex, which is responsible for some of disulfiram’s anti-tumor activity is quite unstable [139]. Thus, this prompted Li et al. to create a nanomedicine that employs copper benzene-1,3,5-tricarboxylate (Cu-BTC), a metal organic framework (MOF) to carry DTC. The combination of Cu-BTC@DDTC with low-dose cisplatin showed significant antitumor effect and a promising safety profile [139]. 

### 3.10. Photodynamic Therapy

Photodynamic therapy (PDT) is a treatment modality that uses a photosensitizer and light of a specific wavelength to generate ROS, such as singlet oxygen, to induce cell death [116,140]. It has been investigated for use in various types of cancer, including melanoma. Studies have incorporated ruthenium complexes and nanoparticles to enhance the efficacy of PDT and minimize side effects [140,141,142]. One study reported that homoligand polypyridyl ruthenium complexes (HPRCs) exhibited increased reactive oxygen species (ROS) generation and decreased dark cytotoxicity, mitigating damage to healthy tissue and demonstrating potential as effective antitumor PDT agents [140]. Concurrently, a systematic review of combined and multidrug therapies based on PDT and photothermal therapy (PTT) showed synergistic effects, particularly in preclinical murine models [116]. Additionally, the utilization of TiO_2_ nanoparticles combined with specific photosensitizers, such as 5,10,15,20-(Tetra-N-methyl-4-pyridyl)porphyrin tetratosylate (TMPyP4), showed promising results, demonstrating improved cell penetration, ROS production, and cancer selectivity, particularly when activated by blue light [141]. Moreover, Ag-doped TiO_2_ nanoparticles used in simulated-daylight photodynamic therapy (SD-PDT) showcased potential in overcoming limitations of conventional PDT, such as severe pain and erythema, by enhancing the daylight response and anti-tumor therapeutic effects [142]. These developments in melanoma treatment indicate a promising future for PDT and PTT-based therapies, especially with the strategic use of nanoparticles and photosensitizers.

### 3.11. Curcumin

Curcumin, a relative of turmeric, is a polyphenolic phytochemical that stimulates reactive oxygen species production and has been shown to induce cell death in eight melanoma cell lines [103,104]. Curcumin induces apoptosis in human melanoma cells through a Fas receptor/caspase-8 pathway independent of p53 and is hypothesized to act through a membrane-mediate mechanism [104] Curcumin has also been shown to block the NF-kappaB cell survival pathway and suppress the apoptotic inhibitor, x-linked inhibitor of apoptosis protein (XIAP). Kocyigit et al. were the first to highlight that ROS may be important in curcumin’s ability to cause DNA damage, cell death, and apoptosis. To test this, they studied the effects of curcumin on murine melanoma cancer cells (B16-F10) and fibroblastic normal cells (L-929) [105]. They found that curcumin caused more DNA damage and apoptosis in melanoma cells than normal cells and also reduced cell viability and mitochondria membrane potential. These effects were linked to the production of ROS, which increased with higher doses of curcumin. One study in human melanoma (A375) cells, showed that curcumin caused oxidative stress by inducing the ROS burst, decreasing GSH, and destroying mitochondria membrane potential (MMP) [143]. Furthermore, apoptosis was induced through ROS-dependent HIF-1α and its downstream proteins [143]. Additionally, curcumin has photoactive properties, and curcumin-based photodynamic therapy has been shown to be effective in reducing the viability of both melanotic (A375) and amelanotic melanoma (C32) cell lines [144]. In Szlasa et al.’s study, photodynamic therapy with curcumin increased the number of apoptotic and necrotic cells compared to incubation with curcumin without irradiation. Although effective, it should be noted that this intervention was not selective towards melanoma cells.

### 3.12. Resveratrol

Resveratrol is an anticancer phytochemical polyphenol that naturally occurs in red grapes [145]. Resveratrol has been shown to downregulate and inactivate Akt/protein kinase B in murine B16F10 and B16BL6 melanoma cells (derived from the former), inhibiting migratory and invasive properties of the malignant cells [106]. Resveratrol also inhibits cell viability and α-MSH-activated matrix metalloproteinase- (MMP-)9 expression and invasion capacity of B16 melanoma cells [107]. Resveratrol has also been shown to decrease NRF2 expression in melanoma cells, inducing increased production of ROS and inhibiting growth and proliferation by downregulating the Bcl-2 protein level and upregulating Bcl-2-related X protein expression [108]. 

Oral, topical, and intraperitoneal resveratrol have all been shown to be effective in melanoma. Oral dosing of resveratrol has been associated with reduced primary tumor volume, Akt expression, and the propensity for metastasis in syngeneic mouse models of melanoma [106]. One investigation evaluating resveratrol-loaded solid-lipid microparticulate topical gel in vitro, showed sustained release profiles, antioxidant properties, tyrosinase inhibition, cytotoxicity, and effective apoptosis in B16F10 melanoma cells [146]. The in-vivo portion of the study in C57BL mice exhibited significant tumor reduction. Another study investigated intraperitoneal injection of resveratrol in immunocompetent mice as a potential therapeutic agent for metastatic melanoma [147]. They found that in vitro growth and proliferation of melanoma cell lines (B16F10, B6, and A375) was significantly suppressed by a dose of 40 μM resveratrol for 72 h. In the mice, in-vivo intraperitoneal injection of 40 mg/kg resveratrol increased mean survival rate and inhibited metastatic lung tumor growth. The lung tumors showed elevated levels of CXCL10 and IFN-γ as well as a reduction in angiogenesis and tumor infiltration by Tregs.

### 3.13. Flavonoids

Flavonoids are polyphenol compounds found in various vegetables, fruits, and other plants [92]. They are well-known for their strong antioxidant properties. Apigenin, diosmin, fisetin, leteolin, quercetin, myricetin, and naringenin are among the commonly studied flavonoids for melanoma treatment [148]. In addition to reducing ROS production, flavonoids have been shown to modulate cell growth, induce apoptosis, and repair DNA. They have also been shown to shrink cancerous tumors and prevent metastasis, and possibly contribute to lower skin cancer mortality rates. The selenocysteine (Sec)-containing mammalian thioredoxin reductase (TRXR) has evolved as a new target for anticancer drug development because TRXR and TRX are overexpressed in many aggressive tumors and the tumor cells are more dependent on the TRX system than normal cells [109,149]. One study suggests that quercetin and myricetin inhibit thioredoxin reductase (TRXR), which is implicated in cellular redox control, halts cell proliferation, and induces apoptosis [109]. Another flavonoid, Naringenin has been shown to block endolysosomal/melanosomal two pore channel 2 (TPC2), thus increasing melanin production while simultaneously reducing MITF (melanocyte inducing transcription factor)-driven melanoma proliferation, migration, and invasion in B16F10 murine melanoma cells [110,111]. Luteolin, has been shown to act as an NRF2 inhibitor in mouse melanoma cells, inhibiting GST and depleting GSH [112]. While flavonoids show promising mechanisms of action in the treatment of melanoma, it should be noted that murine melanoma cells tend to respond better to treatment with flavonoids compared to human melanoma cells, and thus, mouse studies cannot be directly extrapolated to humans [150].

### 3.14. Quassinoids

Similarly, to Flavonoids and Resveratrol, Quassinoids have been shown to downregulate NRF2. Ailanthone, an extract derived from the tree *Ailanthus altissima*, has shown anti-tumor activity by downregulating NRF2 and inducing oxidative stress [113,114,115]. Additionally, low-dose UVA irradiation can act synergistically with brusatol, a *Brucea javanica* plant-derived NRF2 inhibitor, demonstrated increased intracellular ROS, inhibition of melanoma cell proliferation, and induction of cell apoptosis both in vitro and in vivo [151].

## 4. Conclusions

The role of oxidative stress in melanoma is paradoxical in that it promotes tumor initiation but prevents vertical growth and metastasis. As the disease progresses, melanoma employs adaptive mechanisms to decrease oxidative stress in the tumor environment. Thus, agents with antioxidant properties may have the greatest utility in chemoprevention, whereas those with pro-oxidant properties may be better suited for treatment (Table 1).

A promising approach to improving the response of melanoma patients to therapy may involve boosting intracellular ROS production through the use of active biomolecules or by targeting enzymes responsible for managing oxidative stress. Acquired resistance to BRAF/MEK inhibitors can lead to disease relapse, and is thought to at least be in part due to redox metabolic rewiring [14]. Thus, treatment modalities that affect the oxidative stress pathways may prove especially beneficial in drug-resistant melanomas. 

Future investigations should aim to identify and develop novel therapies that can effectively manage oxidative stress in melanoma and further elucidate mechanisms of action of known therapeutic candidates. Combining targeted “oxidative stress pathway therapies” with other treatment modalities such as chemotherapy or immunotherapy, may enhance the response to treatment and provide another option for aggressive or resistant disease. With the standard of personalized medicine on the horizon, identification of reliable predictive biomarkers and tumor characteristics of patients who respond well to antioxidant and/or pro-oxidant-based modalities may be of value. However, clinical studies are first required to ensure efficacy and mitigation of harmful side effects, if any, of antioxidants and pro-oxidants in melanoma therapy.

## Figures and Tables

**Table 1:** Potential preventive and therapeutic agents with antioxidant and pro-oxidant properties and their proposed mechanisms of action.

Intervention	Proposed Mechanism(s) of Action
N-acetylcysteine	Antioxidant properties → potential chemo-preventive agent [89,90] High doses may increase the nuclear translocation of NRF2 → promote metastasis [96]
Selenium	Antioxidant properties → potential chemo-preventive agent [91] May increase tumor growth once disease is present [91]
Carotenoids	Antioxidant properties → no clear preventive benefit [120,121,122,123,124] May accelerate metastasis [97]
Alpha-tocopherol	Antioxidant properties → unclear benefit when taken systemically, but may be chemo-preventive when applied topically [93,120,125,126] May increase migration of melanoma cells and promote metastasis [97]
SOD mimetics	Mimics the activity of SOD enzymes → potential chemo-preventive agent [94] May be useful as adjunct therapy to radiation [128,129]
Taurine	↑ SOD and GPX activity → ↓ ROS [95]
Paraoxonase -2 inhibitors	PON2 exerts antioxidative properties within the mitochondrial respiratory chain → blockade increases oxidative stress [98]
Buthioninesulfoximine	Inhibits gamma-glutamylcysteine synthetase → reduces GSH levels [99]
Disulfiram	SOD inhibitor [100,101,102] Inhibits ubiquitin-proteasome system [137,138,139] Induces ferroptosis mediated by the SLC7A11/GPX4 signaling pathway [139]
Photodynamic therapy	Combines a photosensitizer and light of a specific wavelength to generate ROS, such as singlet oxygen → induces cell death [116,140]
Curcumin	Induces apoptosis through a Fas receptor/caspase-8 pathway and ROS-dependent HIF-1α and its downstream proteins [104,143] Inhibits the NF-kappaB cell survival pathway [104] Induces the ROS burst, decreases GSH, and destroys mitochondria membrane potential [143]
Resveratrol	Downregulation of Akt/protein kinase B → inhibits migratory and invasive properties of the malignant cells [106] Inhibits cell viability and α-MSH-activated MMP-9 expression and invasion [107] Downregulates NRF2 expression [108] Increases CXCL10, IFN-γ, and tumor infiltration by Tregs and reduces angiogenesis → inhibition of metastasis [147]
Flavanoids	Antioxidant properties→ chemo-preventive [92] Quercetin and myricetin: inhibition of TRXR→ induces apoptosis [109] Naringenin: inhibition of TPC2 → increases melanin production and reduces MITF-driven proliferation, migration, and invasion [110,111] Luteolin: inhibits NRF2 → induces oxidative stress [112]
Quassinoids	NRF2 downregulation → induces oxidative stress [113,114,115,151]

NRF2, nuclear factor erythroid 2-related factor 2; SOD, superoxide dismutase; PON-2, paraoxonase -2; GSH, glutathione; HIF-1α, hypoxia inducible factor 1 subunit alpha; NF-kB, nuclear factor kappa B; ROS, reactive oxygen species; α-MSH, alpha-melanocyte stimulating hormone; MMP-9, metalloprotenaise-9; CXCL10, chemokine ligand-10; IFN-γ, interferon gamma; Treg, T-regulatory cells; TRXR, thioredoxin reductase; TPC2, two pore channel 2; MITF, melanocyte inducing transcription factor.
